# Parametric finite element modeling and pose-dependent natural characteristics of a five-axis machine tool

**DOI:** 10.1371/journal.pone.0359727

**Published:** 2026-10-05

**Authors:** Jiamin Zhang, Jiahui Zhang, Ning Lü, Shuaiqi Chen

**Affiliations:** 1 School of Mechanical Engineering, Yangzhou Polytechnic College, Yangzhou, China; 2 Beijing Institute of Fashion Technology, Beijing, China; 3 School of Mechanical Science and Engineering, Huazhong University of Science and Technology, Wuhan, China; China Construction Fourth Engineering Division Corp. Ltd, CHINA

## Abstract

Five-axis machine tools exhibit pose-dependent structural dynamics, but repeated full finite element reconstruction across the workspace is computationally inefficient. We developed a parametric finite element workflow in ANSYS APDL for a five-axis machine-tool analog specimen. The structure was divided into six modules; regular regions were sweep-meshed and irregular regions were represented with quadratic SOLID186 elements using nominal element sizes of 20, 10, and 5 mm. Bearing, ball screw-nut, and linear guideway-slider joints were represented by equivalent springs. Twelve screw-nut and guideway-slider stiffness parameters were calibrated with a genetic algorithm against the first six natural frequencies measured at one reference pose. Movable joint locations were updated from the X-, Y-, Z-, A-, and C-axis pose variables while the identified joint stiffness values were held constant. At the reference pose, the mean and maximum frequency discrepancies between the calibrated model and experiment were 3.29% and 8.78%, respectively. Workspace calculations predicted that the first natural frequency decreased as the beam center of gravity rose, whereas spindle-dominated local modes showed similar trends among working planes. Because calibration and model checking used the same reference-pose data and no multi-pose modal measurements were available, the reported workspace trends should be interpreted as model-based predictions rather than independently validated responses.

## 1. Introduction

Five-axis machine tools, as critical equipment in modern manufacturing, are widely utilized in high-end sectors such as aerospace and automotive industries due to their high degrees of freedom and precision machining capabilities. However, as machining accuracy requirements continue to increase, the dynamic performance of machine tools has become a key factor influencing machining quality [1]. The natural characteristics of the overall machine tool structure, such as natural frequencies and mode shapes, directly affect its dynamic performance, and these characteristics can vary significantly with changes in the machine tool’s pose [2]. Therefore, investigating the pose-dependent natural characteristics of five-axis machine tools is of great significance for enhancing their machining performance.

In recent years, the finite element method [3–6], as a mainstream approach for analyzing machine tool dynamic performance, has been widely applied in the structural modeling and dynamic analysis of machine tools. The analysis of machine tool natural characteristics using the finite element method primarily falls into two categories: high-frequency modal analysis focusing on individual components [7,8] and low-frequency modal analysis targeting the entire machine tool structure [9]. Compared with high-frequency modes, low-frequency modes are more susceptible to excitation. Due to their pose dependency, resonance may occur during machining processes, thereby affecting cutting stability [10,11]. In low-frequency modal analyses that take the whole machine tool as the research object, the mechanical characteristics of the joints must be considered as crucial factors. Joints are key components in machine tool structures for transmitting loads and displacements, connecting different subsystems as well as components within each subsystem. Studies have shown that vibration problems caused by joints account for more than 60% of total machine tool vibration issues [12]; consequently, the mechanical properties of joints must be fully considered when establishing dynamic finite element models of machine tools.

For the dynamic modeling of entire machine tools based on the finite element method, current research on joint modeling primarily employs two main approaches. The first approach involves equivalent modeling of joints using spring elements, where the contact characteristics of different joints are described by varying the spring stiffness in different degrees of freedom. For instance, Yong-sub et al. [13] utilized linear spring elements to equivalently model balls, establishing an equivalent dynamic model for guideway-slider joints. This method was subsequently applied to analyze the natural characteristics of a complete machine tool system, yielding natural frequency errors within 15%. Mao et al. [14] considered the relative motion between fixed joint substructures and the coupling between various degrees of freedom, establishing a general finite element model for fixed joints in machine tools. Using this method to model an MC2000 machining center, the relative error between theoretical and experimental results was less than 10%. The second approach equivalently represents joints as zero-thickness virtual material layers, where the contact characteristics are modified by adjusting the elastic modulus and Poisson’s ratio of the virtual material. For example, Guo et al. [15] established a dynamic model of machine tool joints based on zero-thickness joint interface theory. This method was applied to analyze a five-axis turning-milling composite gantry machine tool, with experimental results showing a maximum relative error of 5.63% for the first six natural frequencies. To improve the accuracy of dynamic modeling for fixed joints in machine tools, Tian et al. [16] proposed a dynamic modeling and analysis method based on the virtual material assumption. A finite element model of the complete machine tool was established using virtual materials, and experimental validation demonstrated good agreement between simulation and experimental results. Beyond equivalent spring and virtual material methods, Brecher et al. [17] introduced multi-point constraints (MPC) through weighted nodes during finite element modeling of machine tool structures. Two additional multi-point constraints were supplemented to more accurately account for the characteristics of ball screw and linear guideway joints. This method was applied to model a three-axis milling machine structure, and experimental results confirmed its capability to reasonably reflect the dynamic characteristics of the overall machine tool structure.

Given the complexity of joints and the variability of material properties, surface roughness, and other attributes, directly determining stiffness parameters presents significant challenges. Deng et al. [18] proposed a joint stiffness configuration method, establishing a finite element model of the complete machine tool that accounts for joint stiffness characteristics, with model accuracy validated through vibration experiments. In this study, fully considering the contact characteristics of various joints in a five-axis machine tool, equivalent dynamic models of each joint were established using spring elements. A genetic algorithm-based inverse identification method was employed to obtain joint contact stiffness, based on which a finite element model of the five-axis machine tool incorporating joint contact characteristics was developed.

Current research on finite element modeling of five-axis machine tools that accounts for pose dependency remains limited, with related studies still facing numerous challenges. On one hand, the dynamic characteristics of five-axis machine tools are influenced by the coupled effects of multiple factors such as structural deformation and gravity under different poses. On the other hand, rapidly updating the model after pose changes presents a major challenge; model updating methods employing coordinate transformations have limitations for analyzing mode shapes. To address the difficulty of efficiently determining the dynamic behavior of multi-axis machine tools as a function of pose variation, Cao et al. [19] adopted an iterative algorithm based on frequency response functions. By iteratively determining joint stiffness parameters while simultaneously updating the finite element model, this approach can reliably predict the pose-dependent dynamic behavior of machine tools. Current investigations of pose dependency often involve establishing multi-body dynamic models [20,21]. To rapidly evaluate and optimize the dynamic behavior of dual-rotary milling heads throughout the entire workspace, Du et al. [22] developed a multi-body dynamic model considering flexible joints. The effects of gravity and cutting forces on flexible joint stiffness at different swing angles were analyzed, and parameterized dynamic equations incorporating both pose parameters and physical parameters were derived. Law et al. [23] established an efficient pose-dependent multi-body dynamic model based on reduced-model substructure synthesis, which effectively simulates the interaction between machine tool structures during machining processes. While these studies provide important theoretical support for analyzing pose-dependent natural characteristics, a balance between solution efficiency and modeling accuracy still requires further optimization.

It can thus be observed that most existing machine tool modeling approaches based on the finite element method neglect the pose dependency arising from multi-axis motions in five-axis machine tools, while also lacking parameter flexibility when dealing with complex structures. This limitation makes it difficult to efficiently and rapidly determine the natural characteristics of five-axis machine tools at multiple poses. Therefore, developing a parametric finite element model capable of accounting for pose dependency is particularly necessary. To address this requirement, the present study proposes a parametric modeling method for five-axis machine tools based on the finite element method, aiming to achieve rapid determination of natural characteristics at multiple poses within the machine tool workspace and systematically analyze the underlying pose-dependent patterns.

Specifically, based on the structural characteristics of five-axis machine tools and utilizing similarity design theory, an analog experimental specimen was designed in this study. This specimen maintains consistency with actual five-axis machine tools in terms of joint characteristics and structural features, effectively mapping the structural properties of real machine tools. First, employing a modular modeling concept, the five-axis machine tool analog specimen system was divided into modules on the ANSYS platform. A mesh partitioning strategy suitable for analyzing pose dependency was designed, and equivalent dynamic models of joints were established. Joint stiffness was inversely identified using a genetic algorithm, and an initial finite element model of the five-axis machine tool system was constructed. Subsequently, the pose transformation scheme was parameterized, enabling rapid model updating following pose changes, thereby establishing a parametric finite element model incorporating both pose dependency and joint contact characteristics. Finally, based on this model, a case study was conducted on the five-axis machine tool analog specimen, achieving rapid determination of natural characteristics at multiple poses within the workspace and exploring the associated pose-dependent patterns.

## 2. Materials and methods

### 2.1. Finite element modeling

To accurately characterize the structural properties of a five-axis machine tool while ensuring both the precision and operability of finite element analysis, a modular finite element modeling approach was proposed based on the dynamic characteristics of the machine tool. In this approach, the complete machine tool system under investigation was first divided into six subsystem modules. A partitioned meshing strategy was then adopted for discretization. Subsequently, equivalent dynamic models of the joints were established. Finally, boundary conditions were introduced to complete the finite element modeling process.

#### 2.1.1. Module division.

The five-axis machine tool was divided into six subsystem modules, including the X-, Y-, and Z-axis feed systems, the spindle system, the worktable system, and the machine bed. Finite element modeling was performed separately for each subsystem module, and the modules were assembled through fixed joints. The subsystem division is illustrated in Fig 1.

**Fig 1:**
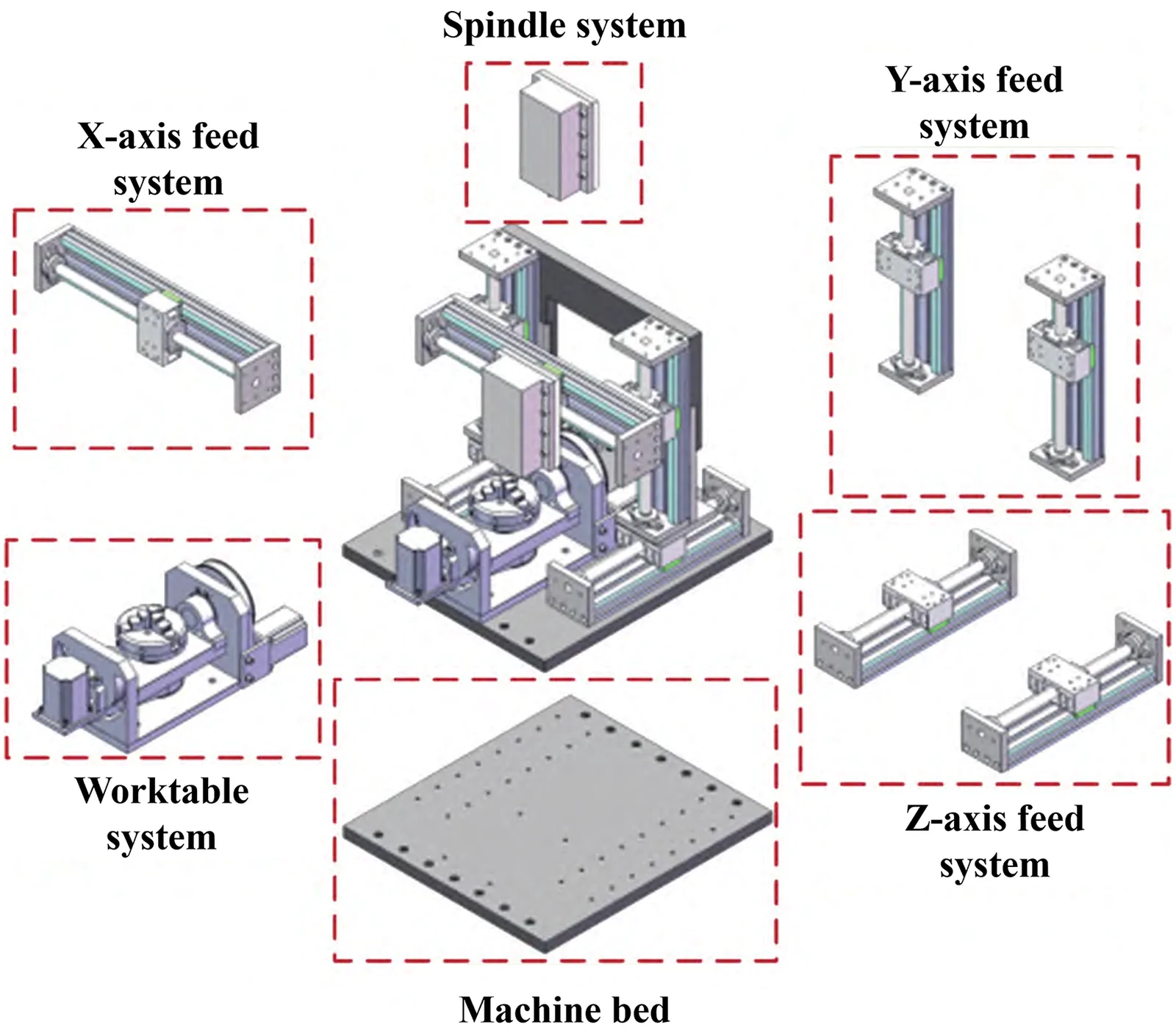
Subsystem division of machine tool simulators. All image content in this figure was created by the authors from their original CAD model.

#### 2.1.2. Meshing strategy.

A mixed meshing strategy was used to balance geometric fidelity and computational cost. All structural subsystems were represented with quadratic SOLID186 elements. Regular regions without circular holes were sweep-meshed (VSWEEP), producing structured hexahedral or wedge-dominant regions where the geometry permitted; irregular regions were free-meshed with tetrahedral SOLID186 elements (MSHAPE,1,3D). Nominal element sizes of 20 mm, 10 mm, and 5 mm were assigned to low-participation large components, intermediate regions, and dynamically important small components or movable-joint neighborhoods, respectively. The final reference-pose model contained 105,949 elements and 209,416 nodes. Fixed interfaces were represented by CONTA174-TARGE170 bonded contact pairs, which permit nonmatching surface meshes. Movable interfaces were connected through spring elements between rigidly coupled reference nodes, as detailed below (Fig 2). A formal mesh-convergence study was not retained in the available analysis record; consequently, the reported discretization is documented for reproducibility but is not presented as mesh-independent. This limitation is considered when interpreting the frequency discrepancies.

**Fig 2:**
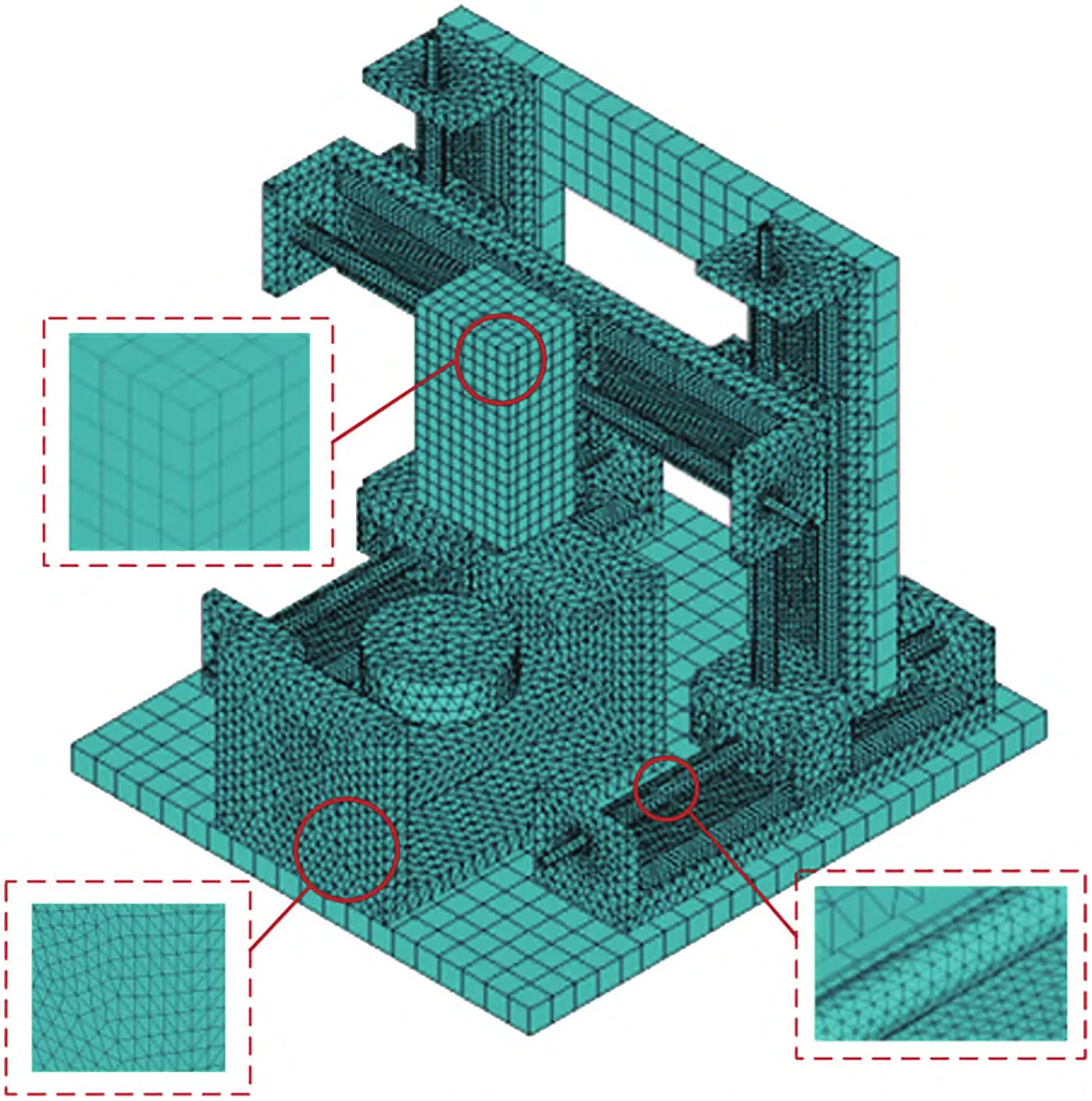
Meshing of machine tool simulators. All image content in this figure was created by the authors from their original ANSYS finite element model.

#### 2.1.3. Equivalent dynamic modeling of joints.

The joints of the five-axis machine tool analog specimen were categorized into four types: fixed joints, bearing joints, ball screw-nut joints, and linear guideway-slider joints. Corresponding equivalent dynamic models were established for each type to ensure the accuracy of the finite element analysis.

#### 2.1.4. Fixed joints.

Fixed joints primarily appear in welded and bolted connections within machine tool structures and typically exhibit high stiffness. In this study, the bonded contact option of the CONTA174 contact element in ANSYS was employed to describe their mechanical characteristics. The CONTA174 element is generally used in conjunction with the TARGE170 element to form a contact pair, meaning that both elements share the same real constant set R. The CONTA174 element is commonly utilized to simulate complex contact behavior between surfaces, and contact can be defined as bonded through appropriate key option settings. The bonded surface-to-surface formulation transfers displacement across the interface without requiring coincident node-to-node meshes; the contact and target surfaces were paired within each assembled fixed joint.

#### 2.1.5. Bearing joints.

For bearing joints, an equivalent dynamic model was established as shown in Fig 3. To reduce geometric model complexity and improve computational efficiency, the bearing cage and rolling elements were neglected. Equivalent springs were created using COMBIN14 elements to simulate the axial and radial stiffness of the bearings. Damping effects were neglected, and the contact stiffness was obtained from product manuals.

**Fig 3:**
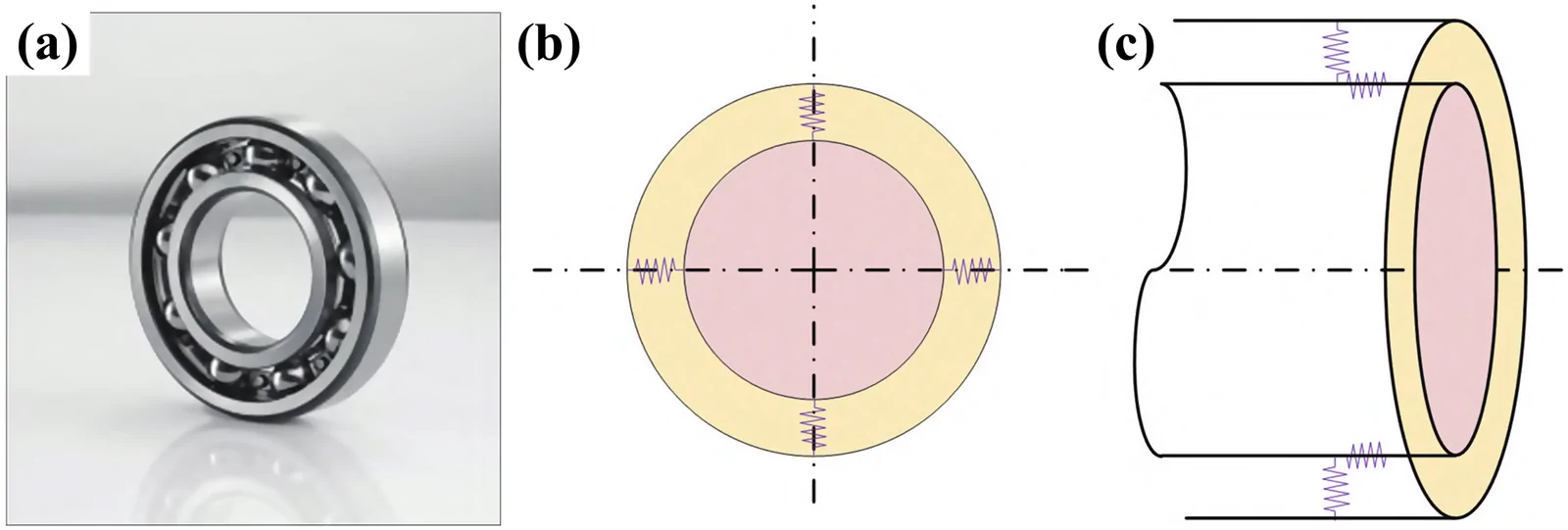
Bearing joint and its mechanical model. (a) Photograph of the physical component; (b) Mechanical model (front view); (c) Mechanical model (left view). The photograph was taken by the authors, and the mechanical schematics were created by the authors.

#### 2.1.6. Ball screw-nut joints.

The ball screw is the most commonly used motion transmission component in machine tools, with its structure illustrated in Fig 4. It mainly consists of the screw shaft, nut, balls, and ball return guides. Through the rolling motion of the balls, the rotational motion of the screw shaft is converted into linear motion of the nut, which is typically employed to accomplish axial feeding along the X-, Y-, and Z-axes of the machine tool. Similar to bearings, the balls and ball return guides were neglected, and COMBIN14 elements were used to simulate axial and radial contact stiffness, without considering damping effects.

**Fig 4:**
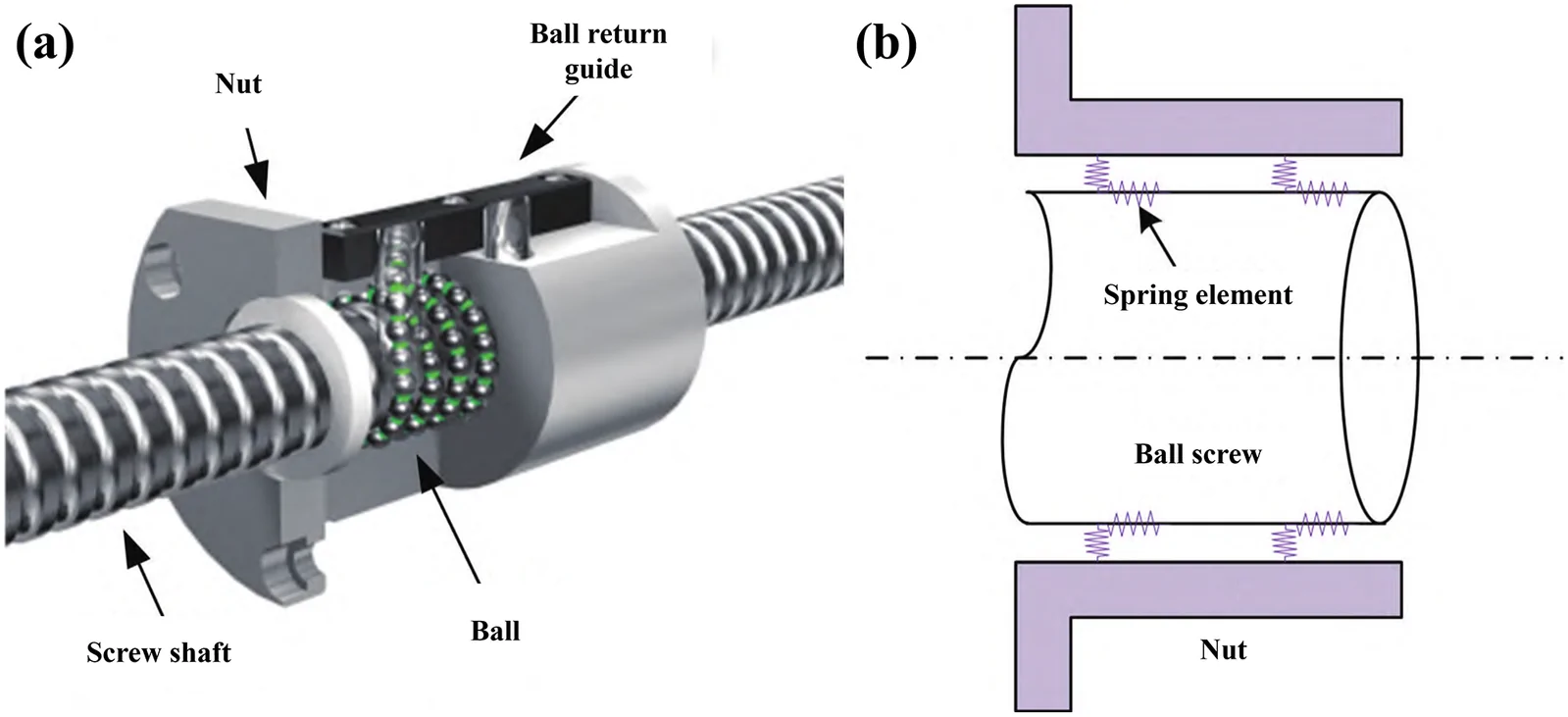
Ball screw-nut joint and its mechanical model. (a) Photograph of the physical component; (b) Mechanical model. The photograph was taken by the authors, and the mechanical schematic was created by the authors.

#### 2.1.7. Linear guideway-slider joints.

Linear guideway-slider joints are responsible for supporting and guiding axial motions and represent one of the most critical joints affecting the dynamic performance of machine tools. To reduce computational effort, the rolling grooves of the guideway and slider were neglected, and these components were simplified into regular rectangular prisms. The contact stiffness characteristics between the guideway and slider were equivalently represented by three sets of spring elements uniformly distributed along the feed direction, establishing an equivalent dynamic model as shown in Fig 5.

**Fig 5:**
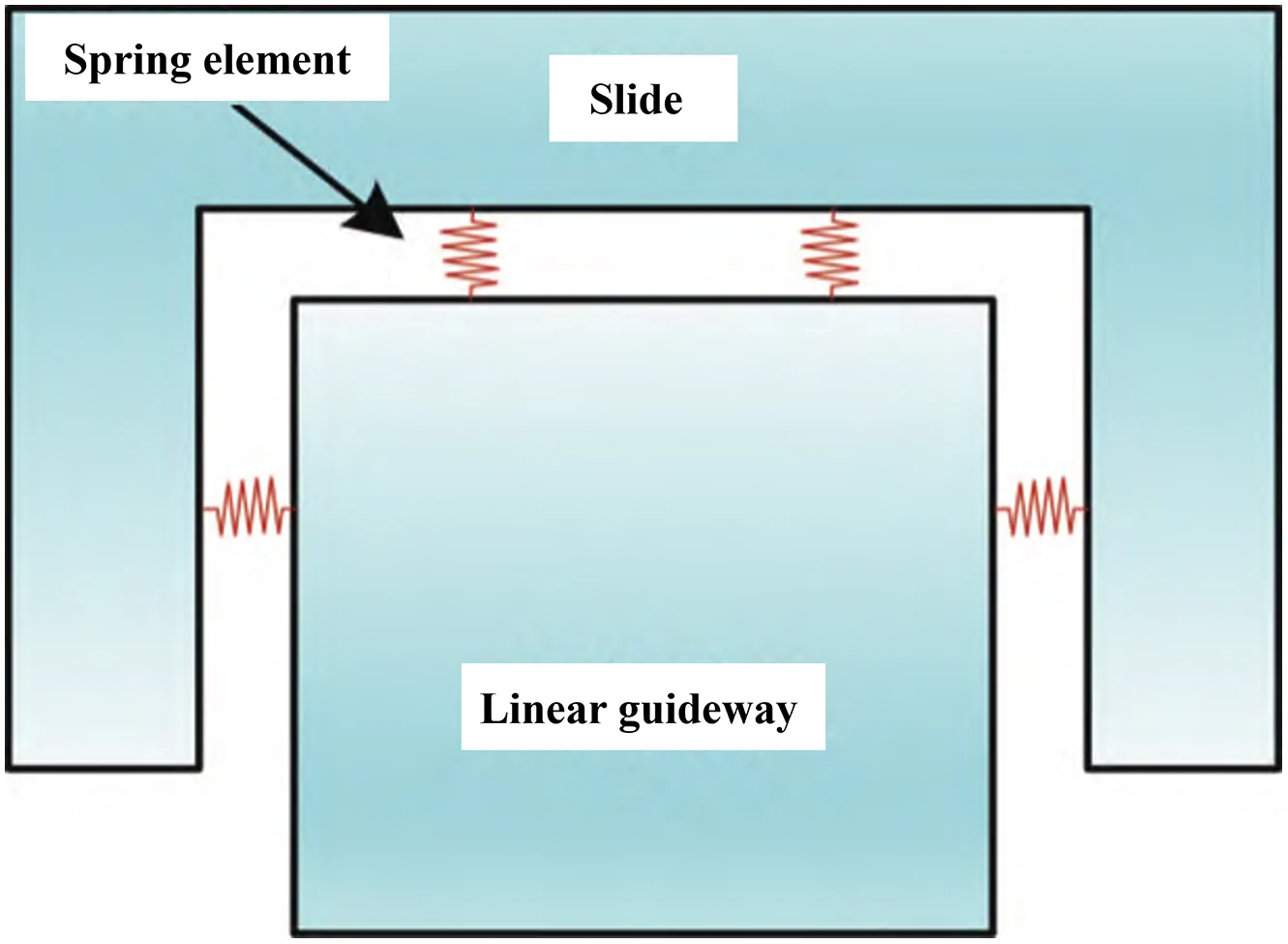
Linear guide−slider joint.

#### 2.1.8. Joint parameter identification.

In the equivalent dynamic models of joints, spring elements were employed for connection, and the contact characteristics of joints were simulated using spring stiffness in each degree of freedom. However, the contact characteristics of joints are highly variable and not easily determined directly. To address this issue, a genetic algorithm was adopted for stiffness identification of the ball screw-nut and linear guideway-slider joints. Through the global search capability of the genetic algorithm, the joint stiffness parameters requiring identification were automatically optimized to minimize the error between simulation and experimental results, thereby obtaining more accurate joint stiffness values. The twelve optimized variables were the axial and radial stiffnesses of the X-, Y-, and Z-axis screw-nut joints and the normal and tangential stiffnesses of the corresponding guideway-slider joints. Bearing stiffness was prescribed from the product manual and was not optimized.

Co-simulation was implemented by calling ANSYS from MATLAB, and the fitness function was defined as the average error of the first six natural frequencies obtained from modal experiments on the five-axis machine tool analog specimen and those solved by ANSYS:


$$\begin{array}{c} Min\;F(x)=\frac{\text{1}}{\text{6}}\times \sum_{i=\text{1}}^{\text{6}}\left|\frac{f_{Ai}-f_{Ei}}{fEi}\right|,x_{L}\leq x\leq x_{U}\;\; \end{array}$$ (1)

where $f_{Ai}$ represents the *i*-th natural frequency obtained from ANSYS simulation, $f_{Ei}$ denotes the *i*-th natural frequency obtained from experiments, and $x_{U}$ and $x_{L}$ are the upper and lower bounds of the contact stiffness parameters, respectively. Through iterative searching, the optimal solution for the joint stiffness parameters was continuously pursued. The specific identification process is illustrated in Fig 6. The fully fixed constraints applied to the machine bed are illustrated in Fig 7. When inverse identification experiments cannot be performed, joint stiffness values for similar machine tools or structures can be obtained from existing literature and engineering experience as preliminary estimates. Alternatively, theoretical calculation methods such as Hertz contact theory and Yoshimura’s integral method may be employed to determine joint stiffness.

**Fig 6:**
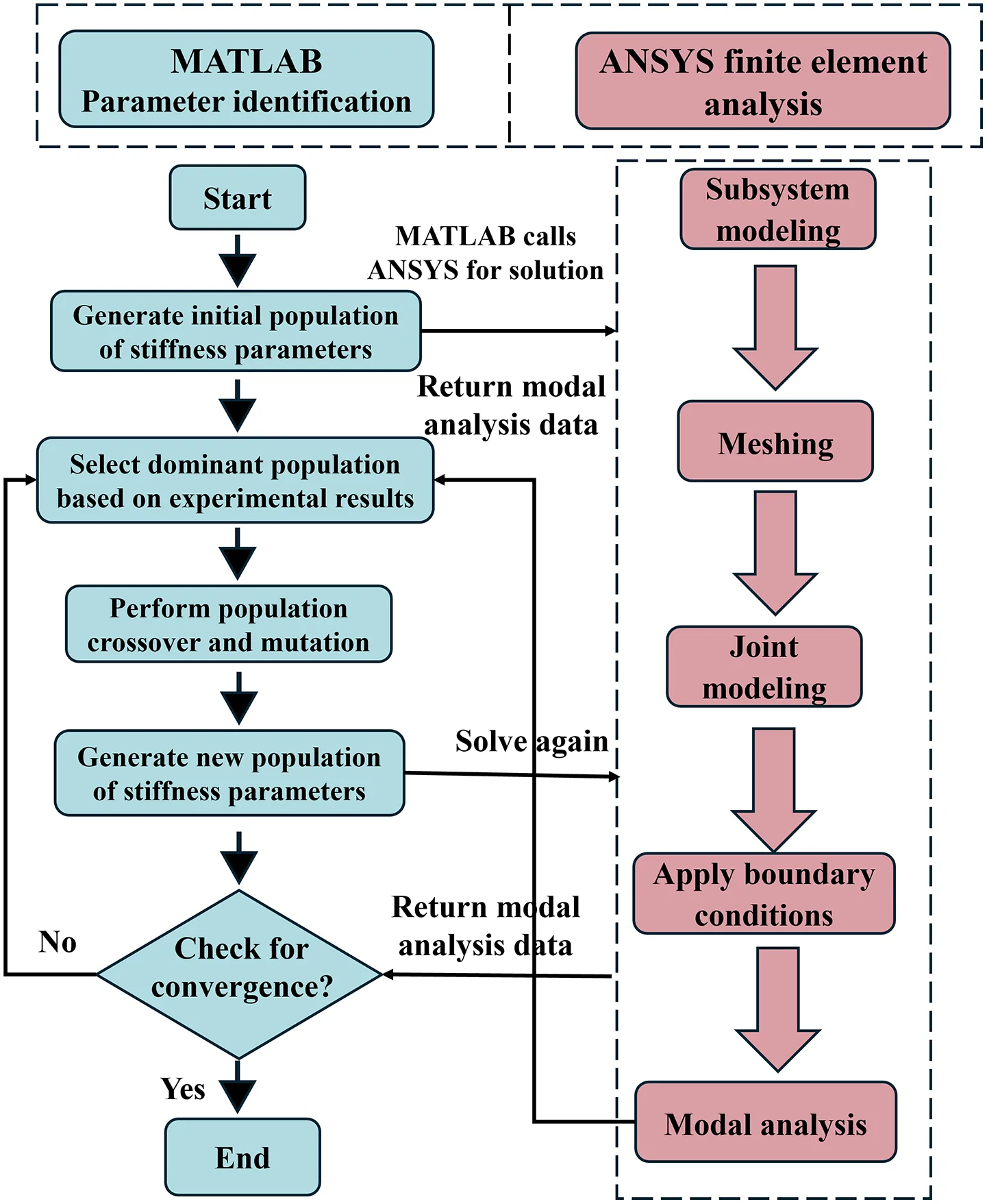
Identification process of contact stiffness.

**Fig 7:**
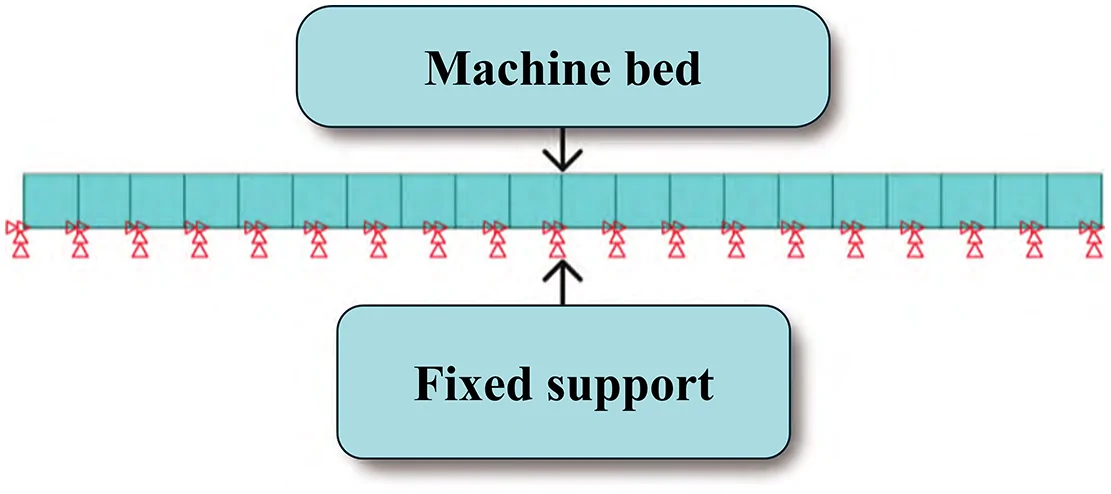
Fully fixed constraints.

### 2.2. Parametric modeling

For the analysis of pose-dependent natural characteristics of five-axis machine tools, a parametric modeling method based on the finite element method was proposed to achieve rapid model updating following pose changes. By identifying key driving variables and establishing parametric equivalent spring models to simulate movable joints, accurate modeling suitable for analyzing pose-dependent natural characteristics of the machine tool was accomplished.

#### 2.2.1. Classification of parametric variables.

In the parametric modeling process of five-axis machine tools, the selection of driving variables significantly influences the adaptability and accuracy of the model. Five-axis machine tools possess complex structures, comprising multiple motion axes and their associated structural components, whose dynamic characteristics vary with changes in pose. To effectively characterize the pose-dependent dynamic behavior, the parametric variables in this study were classified into driving variables (controlled variables) and uncontrolled variables.

#### 2.2.2. Driving variables.

Driving variables included the pose parameters of the motion axes. The five-axis machine tool under investigation consisted of three linear motion axes (X, Y, and Z) and two rotary motion axes (A and C). The positions of the linear motion axes determined both the relative positions between subsystems and the center-of-gravity positions of the subsystems themselves, whereas the rotary axes determined the orientation of the workpiece-table assembly. For the analog specimen, the interference-constrained coordinate ranges used in the parametric calculations were X = 0–300 mm, Y = 200–350 mm, and Z = 100–300 mm. The A- and C-axis variables were implemented in the parametric formulation; the planar calculations reported in Fig 14 used A = 0 degrees and C = 0 degrees. These pose coordinates were designated as driving variables for model reconstruction and modal solution at each sampled configuration.

#### 2.2.3. Uncontrolled variables.

To describe the prerequisite conditions or environmental conditions required for implementing parametric modeling, in addition to the pose parameters of the machine tool motion axes, the geometric assembly parameters of the machine tool structure, as well as key structural and material parameters, were modeled as uncontrolled variables. These uncontrolled variables were determined based on the actual five-axis machine tool configuration. Specifically, geometric parameters included the feed-direction length of the slider, the feed-direction length of the nut holder, the length of the ball screw, and the assembly spacing between the slider and nut holder. Material parameters encompassed the density, elastic modulus, and Poisson’s ratio of both the nut holder and the ball screw.

#### 2.2.4. Parameterization of movable joints and model updating.

To accurately characterize the dynamic behavior of the five-axis machine tool under different poses, dynamic updating of the finite element model was required. As the pose changed, the positions of movable joints (including the ball screw-nut and linear guideway-slider joints) also varied. Therefore, parameterized movable joints needed to be established to accurately represent the complete machine tool model at different poses. In the present implementation, the identified joint stiffness values were held constant throughout the workspace. Pose dependence was introduced through changes in component coordinates, relative assembly positions, mass distribution, and the locations of the equivalent joint springs; load- or pose-induced changes in contact stiffness were not modeled.

#### 2.2.5. Parameterization of movable joints.

For movable joints, an equivalent spring method employing rigidly coupled massless nodes was proposed to achieve adaptive adjustment of spring positions for different poses, thereby ensuring accurate establishment of the complete machine tool model at various poses. Taking the ball screw-nut joint shown in Fig 8 as an example, a massless node (MASS21 element) was first created at the center of the outer cylindrical contact surface of the ball screw. Subsequently, the nodes on the contact surface were rigidly coupled with the newly created massless node using the CERIG command. The same procedure was performed on the inner cylindrical surface of the nut. Finally, a spring (COMBIN14 element) was established between the two massless nodes. Since the coordinates of the newly created massless nodes were parameterized, their positions varied with the positions of the connected movable components. The reference nodes were assigned no mass or inertia. CERIG imposed kinematic compatibility between each interface surface and its reference node, while the COMBIN14 element transferred the calibrated directional force-displacement relation between the two reference nodes. This construction avoids directly tying dissimilar surface meshes and keeps the coupling definition unchanged when the reference-node coordinates are updated.

**Fig 8:**
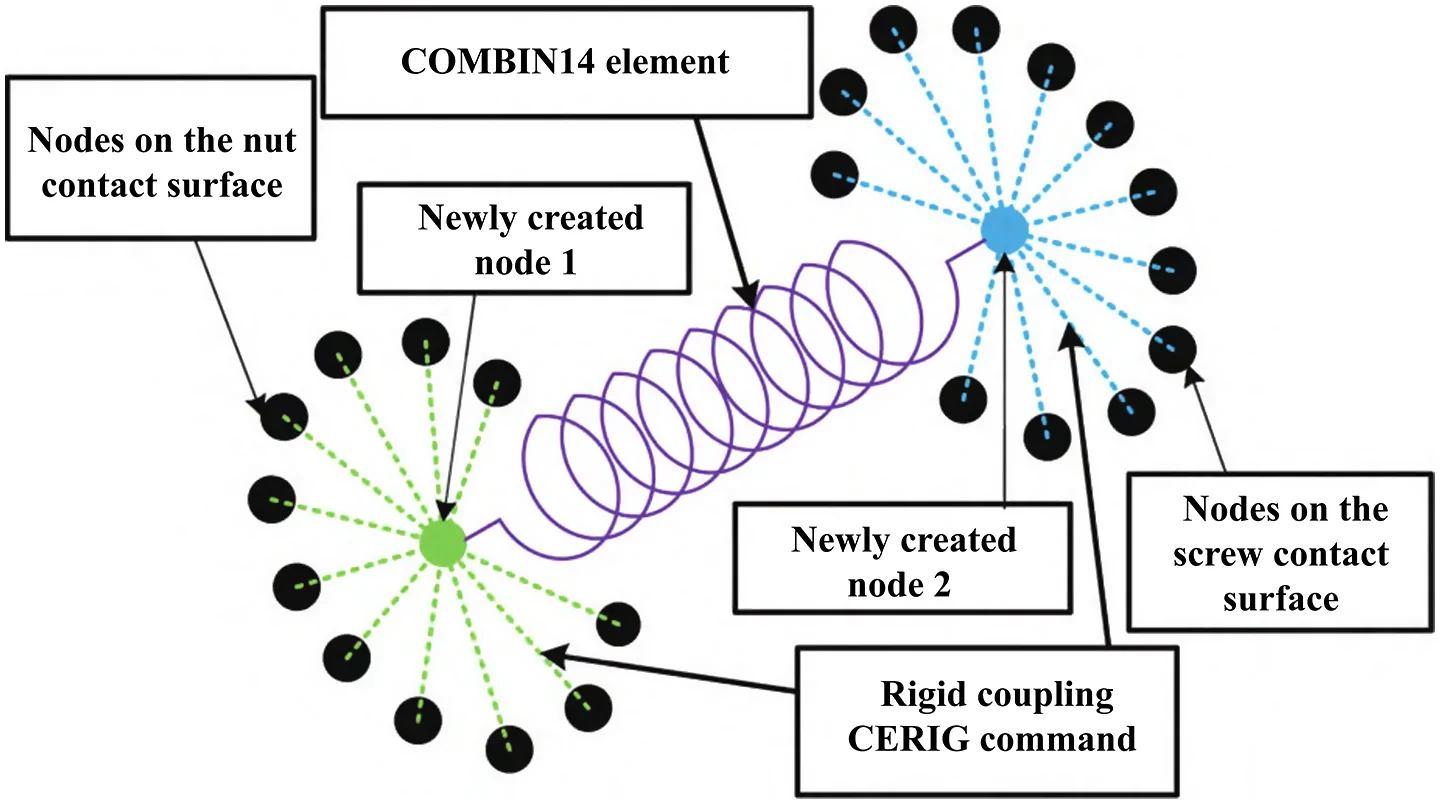
Rigid coupling of nodes.

#### 2.2.6. Model updating method.

To achieve rapid determination of the natural characteristics of the five-axis machine tool under different poses, a parametric model updating method based on a local updating mechanism was proposed in this study. The overall workflow of this method was as follows. First, the modeling process was modularized and controlled through subroutines in ANSYS, which were mainly divided into four subroutines: driving variable definition, modeling, solving, and model updating. Using the *DO loop statement, cyclic calculations for multiple poses were realized. Upon completion of the calculation for one pose, the system determined whether model updating was required. If new pose parameters were present, the updating process was initiated. The first step of model updating involved the deletion of the finite element model corresponding to the previous pose. To ensure that the deletion process did not affect the fixed components in the model (such as the machine bed and column, which did not change with pose variation), a selective deletion strategy was adopted. Only the geometric models and finite element models associated with moving components (such as the spindle, nut holder, and sliders) were removed. The deletion operation was performed strictly following the sequence of “elements first, then nodes” and “bodies first, then points”: that is, the elements and nodes in the finite element model were cleared first, followed by the bodies, areas, lines, and key points in the geometric model. Subsequently, the system read a new pose parameter table, which was imported from an external text file as an array using the *VREAD command. Based on the new pose parameters, the coordinate positions of the movable components were recalculated. For example, for the Z-axis slider system, the position coordinates of the slider and nut holder under the new pose were derived through geometric relationships, thereby determining the new node positions for the spring elements in the movable joints (such as the linear guideway-slider and ball screw-nut joints). After completing the coordinate updates, the geometric model was re-established and meshed. Simultaneously, the spring elements for the movable joints were re-established using the rigidly coupled massless node method. Following the modeling phase, the solving subroutine was re-entered, where the natural frequencies and mode shapes under the new pose were solved using the Lanczos method. The results were then extracted using the *GET command and stored in designated files using the *MWRITE command. It should be noted that model deletion did not involve removing the entire model, but rather only those parts whose positions changed with the pose parameters, such as the spindle, nut holder, and sliders. Components whose positions did not change with pose variation, such as the machine bed, could be retained to improve modeling efficiency (as shown in Fig 9). The deletion of the previous pose model was performed following the sequence of “elements first, then nodes” and “bodies first, then points”: that is, the elements and nodes of the finite element model were cleared first, followed by the bodies, areas, lines, and key points of the geometric model. Subsequently, the new pose parameters were read, and other related parameters, such as the coordinates of movable components, were recalculated based on the new pose parameters. Then, a new geometric model was created and meshed, and the spring elements for movable joints were re-established. Finally, the natural frequencies and mode shapes were solved.

**Fig 9:**
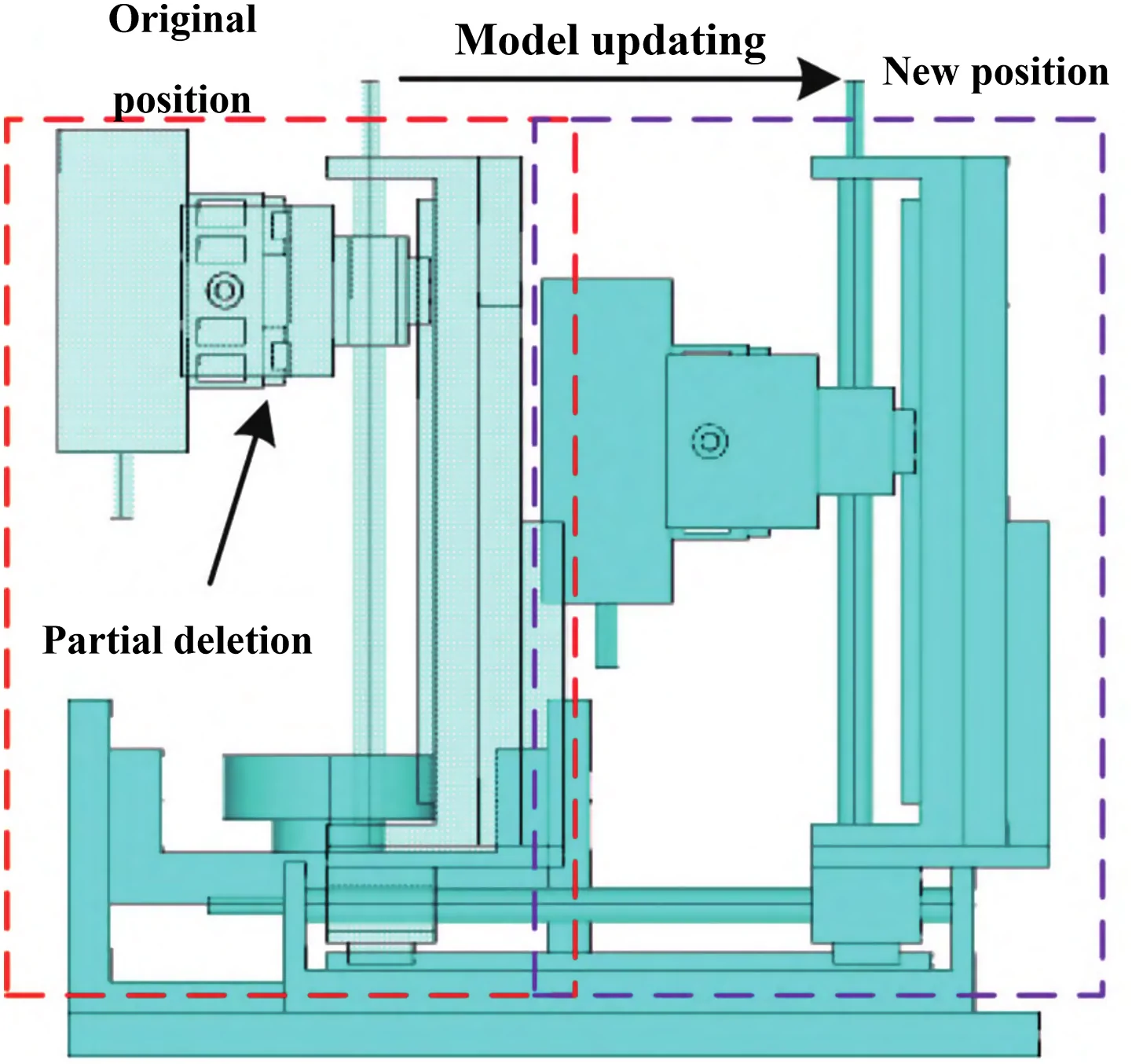
Schematic of model update. All image content in this figure was created by the authors from their original CAD model.

Taking the Z-axis guideway-slider feed shown in Fig 10 as an example, the process of calculating the coordinates of related movable components based on new pose parameters is described. In Fig 10, *D*_*z*_ represents the Z-axis feed displacement, which was a driving variable. *L*_*1*_ and *L*_*2*_ denote the assembly spacings between the nut holder and the left and right ends of the slider, respectively. LLM and LHM represent the lengths of the nut holder and the slider, respectively, which were determined by component dimensions. From the geometric relationships, it can be derived that:

**Fig 10:**
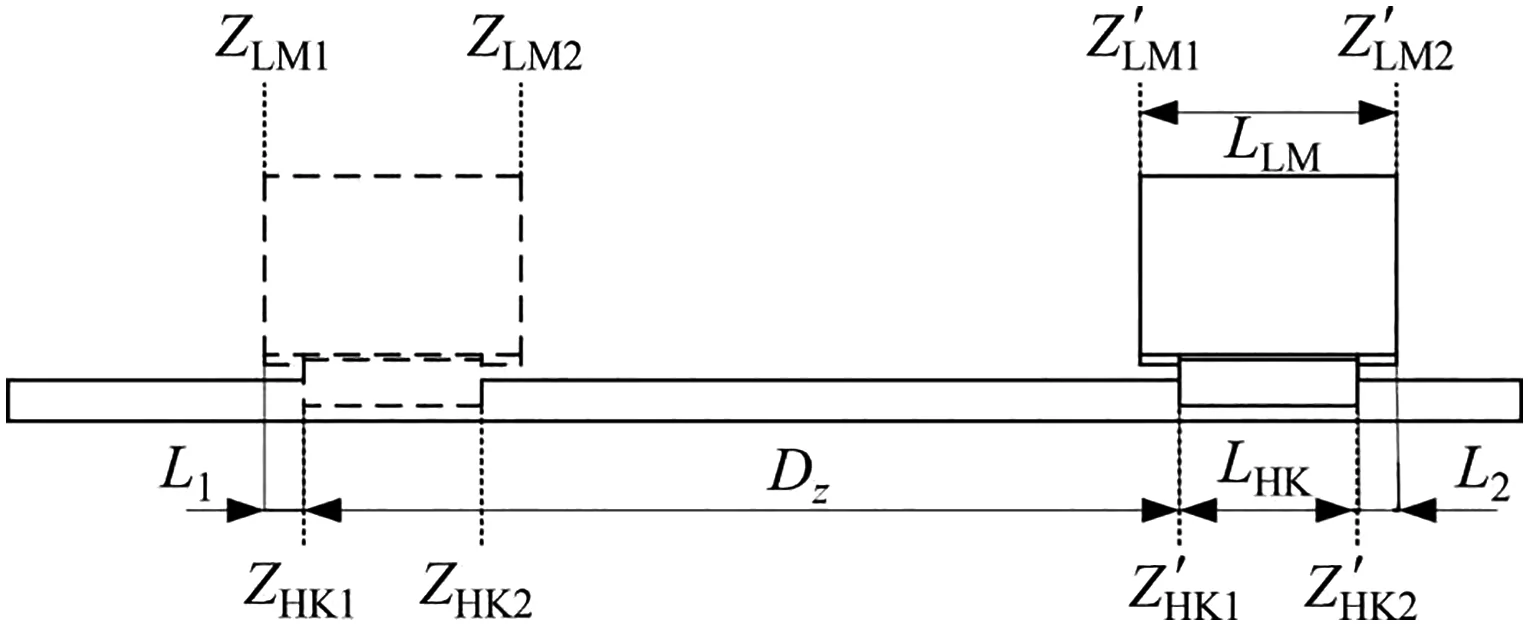
Schematic of Z-axis slide feed.


$$\begin{array}{c} L_{\text{LM}}=L_{\text{1}}+L_{\text{HK}}+L_{\text{2}}\;\;\; \end{array}$$ (2)

Let *Z*_HK1_, *Z*_HK2_, *Z*_LM1_, and *Z*_LM2_ represent the coordinates of the two ends of the slider and nut holder in the Z-axis feed direction, respectively. Assuming *Z*_HK1_ = z and taking *Z*_HK1_ as the reference point, the remaining three coordinates and the four new coordinates after feeding could be obtained through geometric relationships:


$$\begin{array}{c} Z_{\text{HK2}}=z+L_{\text{HK}}\;\;\;\; \end{array}$$ (3)

$$\begin{array}{c} Z_{\text{LM1}}=z-L_{\text{1}}\;\;\;\;\; \end{array}$$ (4)

$$\begin{array}{c} Z_{\text{LM2}}=Z_{\text{LM1}}+L_{LM}=z-L_{\text{1}}+L_{\text{LM}}\;\; \end{array}$$ (5)

$$\begin{array}{c} {Z\prime }_{\text{HK1}}=Z_{\text{HK1}}+D_{\text{z}}=z+D_{\text{z}}\;\;\; \end{array}$$ (6)

Using a subroutine programming approach, rapid updating of the five-axis machine tool model following pose changes was implemented in ANSYS. This was divided into four subroutines: driving variable definition, modeling, solving, and model updating. Using the *DO loop statement, cyclic calculation of natural characteristics at multiple poses was achieved. After the calculation for the previous pose was completed, the model updating subroutine cleared the model sequentially using commands such as EDELE, NDELE, and VDELE. The driving variable definition subroutine read the new pose parameters, and the coordinates of movable components such as *Z*_*HK*_ were defined by the aforementioned geometric relationships. Since the newly created node coordinates of movable joints were related to the coordinates of movable components, the movable joint coordinates were defined simultaneously. Subsequently, the modeling subroutine established the finite element model for the new pose using the new parameters. Finally, the solving subroutine solved for natural frequencies and mode shapes, with data extracted using the *GET command and stored using the *MWRITE command. The pose parameters were determined from a pre-established pose parameter table, which was written into a.txt text file. The pose parameter table from the text file was read into ANSYS as an array using the *VREAD command, making it available for the driving variable definition subroutine.

It should be noted that the *VREAD and *MWRITE commands cannot be entered interactively directly in ANSYS; they require the creation and execution of macro files, such as *CREATE, READ and/INPUT, READ.

### 2.3. Case study and experimental setup

A case study was conducted on the five-axis machine tool analog specimen. A parametric finite element model of the analog specimen was created, and the analysis of its pose-dependent natural characteristics was ultimately accomplished.

#### 2.3.1. Machine-tool specimen and finite element implementation.

The five-axis machine tool analog specimen adopted a moving beam and moving column gantry structure. It was composed of a solid steel plate simulating the machine bed, five ball screw slide tables, a motor spindle, and a dual rotary table assembly. The solid steel plate simulating the machine bed contained through-holes with a diameter of 10 mm for installation using M8 bolts. The ball screw slide tables simulated the three linear feed motion axes, consisting of four units with a 200 mm stroke (for the Y- and Z-axes) and one unit with a 300 mm stroke (for the X-axis). The primary materials used were steel and aluminum alloy. The material densities, elastic moduli, and Poisson’s ratios are presented in Table 1, while the materials and dimensional specifications of the machine bed and key components are provided in Table 2.

**Table 1:** Material parameters of 5-axis machine tools.

Material	Density (kg/m^3^)	Elastic Modulus (GPa)	Poisson’s Ratio
Steel	7.85 × 10^3^	206	0.26
Aluminum alloy	2.91 × 10^3^	69.7	0.33

**Table 2:** Materials and dimensions of key components.

Component	Material	Length × Width × Height (mm)
Machine bed	Steel	400 × 450 × 20
Spindle	Steel, aluminum alloy	80 × 40 × 150
Worktable system	Steel, aluminum alloy	148 × 242 × 145
Slider	Aluminum alloy	90 × 50 × 35

Based on the finite element and parametric modeling methods described above, a finite element model of the five-axis machine tool analog specimen was established. Components with negligible deformation effects, such as motors, were modeled as concentrated mass points. Small features including holes, chamfers, and fillets were ignored. A comparison between the simplified geometric model and the actual three-dimensional model is illustrated in Fig 11. In ANSYS, Solid186 solid elements were selected for modeling each subsystem to ensure satisfactory model accuracy. Following the previously described meshing strategy, element sizes of 20 mm, 10 mm, and 5 mm were adopted. A total of 105,949 elements and 209,416 nodes were generated. For the joints, COMBIN14 elements were employed to simulate spring stiffness, while damping effects were neglected. Fixed supports were applied to the bottom surface of the machine bed as boundary conditions. Modal analysis was performed using the Lanczos method. The element formulation and quantitative mesh description are reported in Section 2.1.2; the values are repeated here only to define the reference-pose implementation.

**Fig 11:**
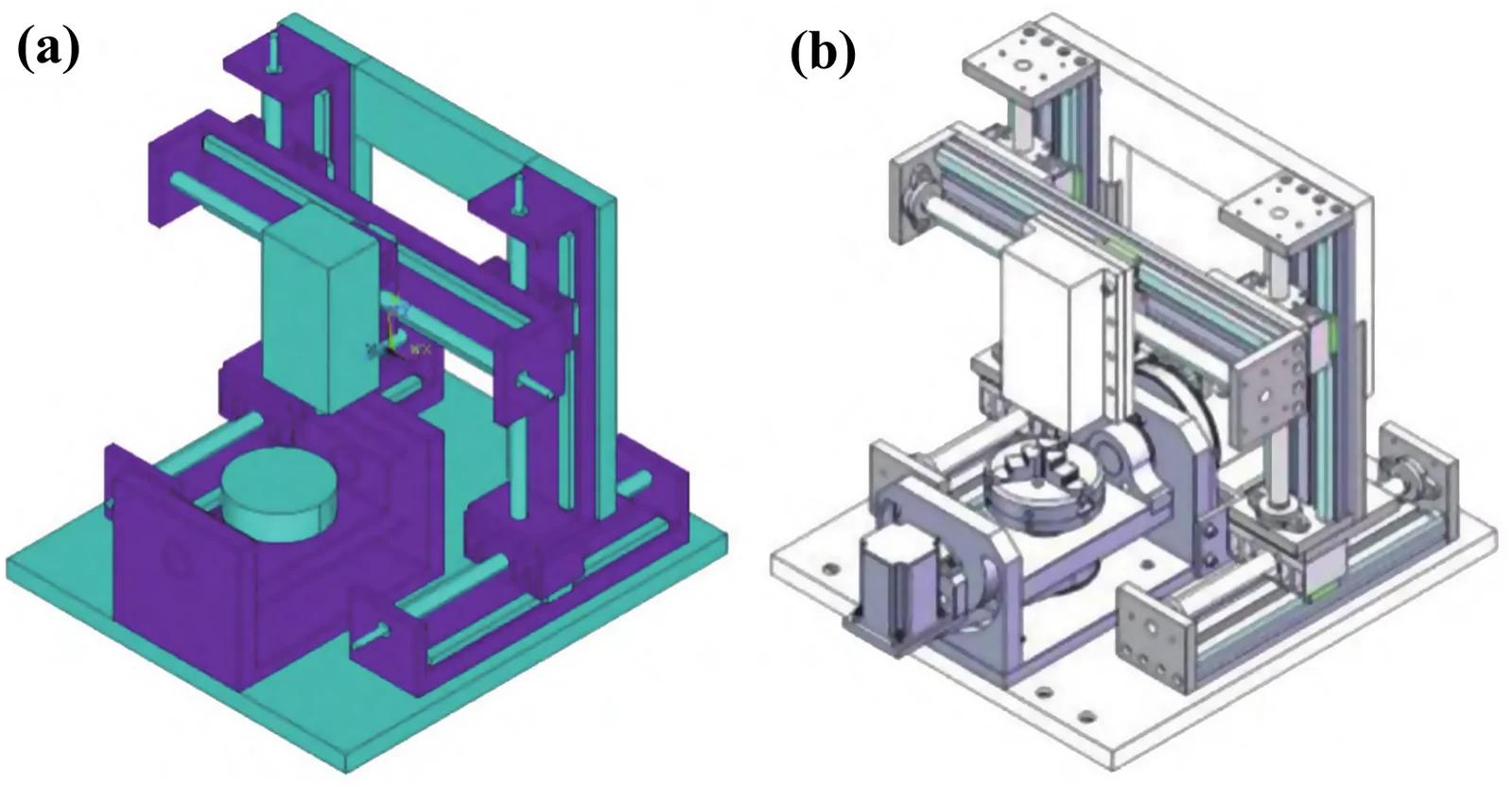
Comparison of models. All image content in this figure was created by the authors from their original CAD and ANSYS models.

#### 2.3.2. Modal test and reference-pose calibration.

Modal experiments were conducted on the five-axis machine tool analog specimen at a specific pose to determine its natural frequencies and mode shapes, providing reference data for the inverse identification of joint stiffness and the reference-pose calibration and model checking. The experimental setup is illustrated in Fig 12, which mainly included PCB356A01 miniature triaxial acceleration sensors, a PCB 086C01 modal impact hammer, an LMS 16-channel data acquisition front-end controller, and an LMS Test.Lab notebook workstation. Due to the complex structure of the five-axis machine tool, a single-point excitation and multi-point vibration pickup method was employed. The excitation position of the impact hammer was kept constant, while the sensor positions were varied to collect vibration response signals at different locations. Considering the actual mode shapes and assembly constraints of the five-axis machine tool, measurement points were arranged on the overall structure. A measurement point model was established in LMS Test.Lab software, omitting the machine bed which did not participate in vibration in the ANSYS simulations. The arrangement of measurement points on components with insignificant vibration, such as the Z-axis feed system and Y-axis ball screw, was appropriately simplified. The final measurement point model is shown in Fig 13, consisting of a total of 235 sensor measurement points, with the excitation point located at position 131. The reference pose used for both stiffness calibration and the internal model check was X = 150 mm, Y = 275 mm, Z = 200 mm, A = 0 degrees, and C = 0 degrees.

**Fig 12:**
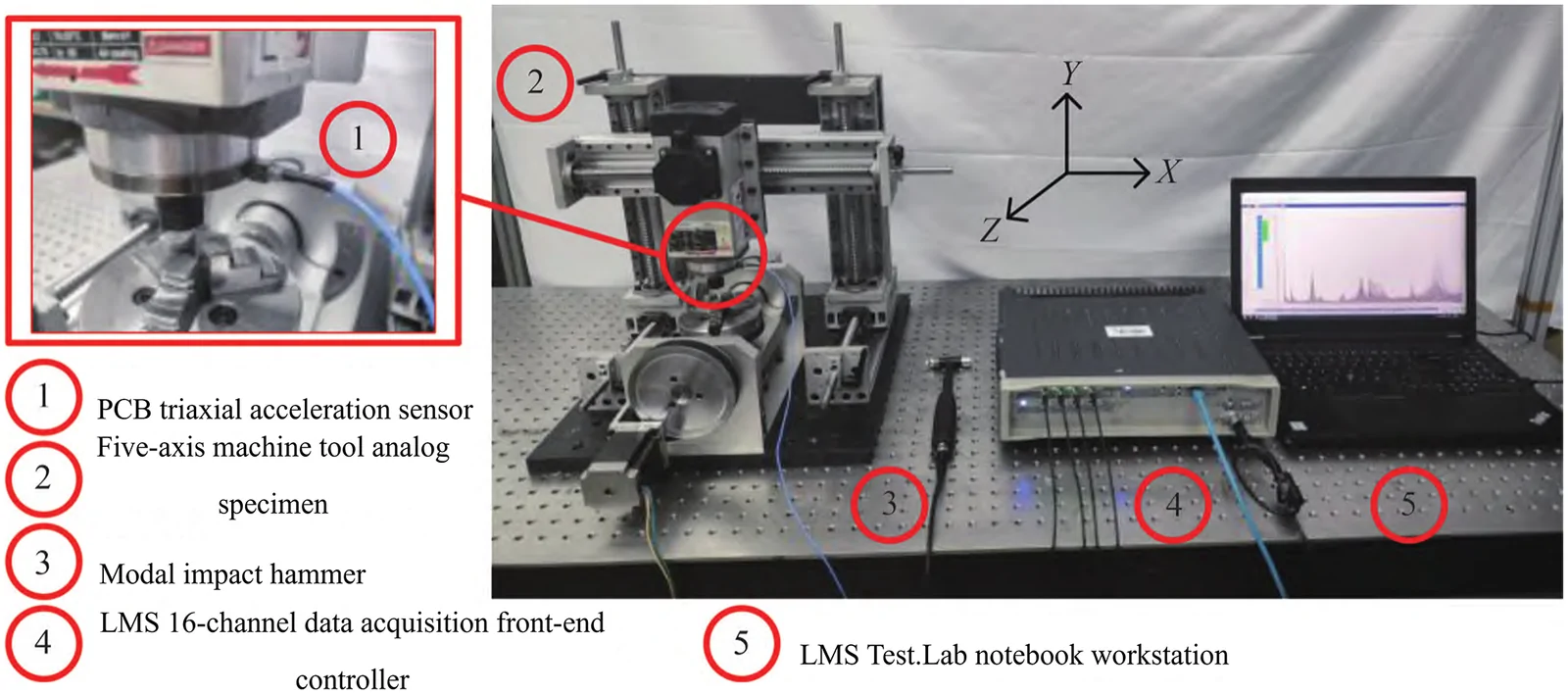
Testing system. The photograph was taken by the authors.

**Fig 13:**
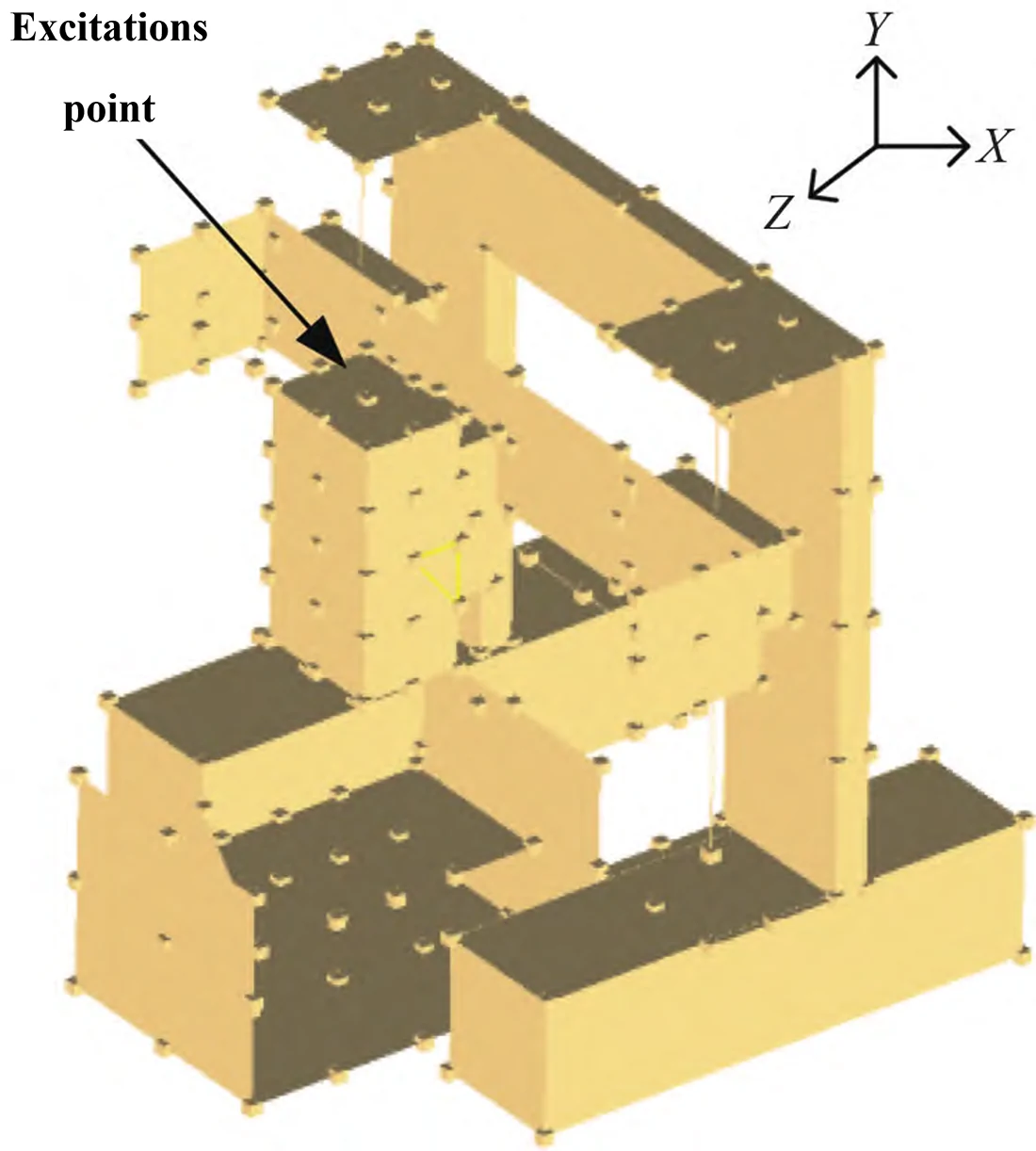
Arrangement of measurement point. All image content in this figure was created by the authors from their original test model.

Based on simulation results, the test bandwidth was set to 1024 Hz with a resolution of 2 Hz and a sampling time of 0.5 s. Two impacts were performed at each measurement point, and the results were averaged to ensure a satisfactory signal-to-noise ratio for the experiment. The PolyMAX algorithm in LMS Test.Lab software was employed to extract mode shapes. Peaks in the frequency response functions corresponding to identical mode shapes were considered as the same modal order, and the natural frequencies of the first six modes were extracted. Using these natural frequencies as a benchmark, the joint stiffness parameters of the finite element model were inversely identified through a genetic algorithm. The crossover probability was set to 0.7, and the mutation probability was set to 0.05 to enhance the algorithm’s ability to escape local optima without significantly reducing convergence. No error threshold was specified, and the number of iterations was set to 100. The identified joint stiffness parameters are presented in Table 3. It should be noted that the bearing joint stiffness was not included in the identification process; instead, the bearing contact stiffness was determined by directly consulting the bearing product manual [18], specifically 5.00 × 10^6^ N/mm. The two impacts at each response location were averaged before modal parameter estimation. The present revision reports the acquisition bandwidth, frequency resolution, record duration, impact count, sensor layout, and PolyMAX estimator settings available in the study record. Representative FRF and coherence spectra were not retained in the manuscript source package, and the experimental mode-shape vectors required for a quantitative autoMAC matrix were not available; therefore, these diagnostics are not reconstructed or inferred.

**Table 3:** Joint stiffness values and parameter treatment.

Joint	Direction	Final stiffness (N mm − 1)	Parameter source/ treatment
Z-axis screw-nut	Radial	1.36 × 10^4^	Genetic-algorithm optimized at the reference pose
	Axial	9.26 × 10^3^	Genetic-algorithm optimized at the reference pose
Z-axis guideway-slider	Normal	9.83 × 10^3^	Genetic-algorithm optimized at the reference pose
	Tangential	1.92 × 10^4^	Genetic-algorithm optimized at the reference pose
Y-axis screw-nut	Radial	3.01 × 10^3^	Genetic-algorithm optimized at the reference pose
	Axial	1.78 × 10^4^	Genetic-algorithm optimized at the reference pose
Y-axis guideway-slider	Normal	7.86 × 10^4^	Genetic-algorithm optimized at the reference pose
	Tangential	1.30 × 10^5^	Genetic-algorithm optimized at the reference pose
X-axis screw-nut	Radial	2.83 × 10^3^	Genetic-algorithm optimized at the reference pose
	Axial	1.45 × 10^4^	Genetic-algorithm optimized at the reference pose
X-axis guideway-slider	Normal	1.87 × 10^4^	Genetic-algorithm optimized at the reference pose
	Tangential	1.05 × 10^4^	Genetic-algorithm optimized at the reference pose
Bearing	Lumped axial/radial	5.00 × 10^6^	Product manual; prescribed and not optimized

## 3. Results

### 3.1. Reference-pose model comparison

After the final stiffness values in Table 3 were incorporated, the first six natural frequencies were calculated and compared with the same reference-pose modal dataset used in calibration. The mean absolute relative frequency discrepancy was 3.29%, and the largest discrepancy was 8.78% for the third mode (Table 4). The qualitative mode-shape comparison is shown in Fig 14. Because the experimental dataset used for stiffness calibration was also used for this comparison, these results constitute an internal model check rather than independent validation.

**Table 4:** Reference-pose experimental and simulated natural frequencies.

Experimental natural frequency/Hz	Simulated natural frequency/Hz	Relative error/%
16.53	16.52	0.06
62.50	64.93	3.89
129.12	140.46	8.78
172.60	172.50	0.06
285.85	286.12	0.09
342.54	318.98	6.88

**Fig 14:**
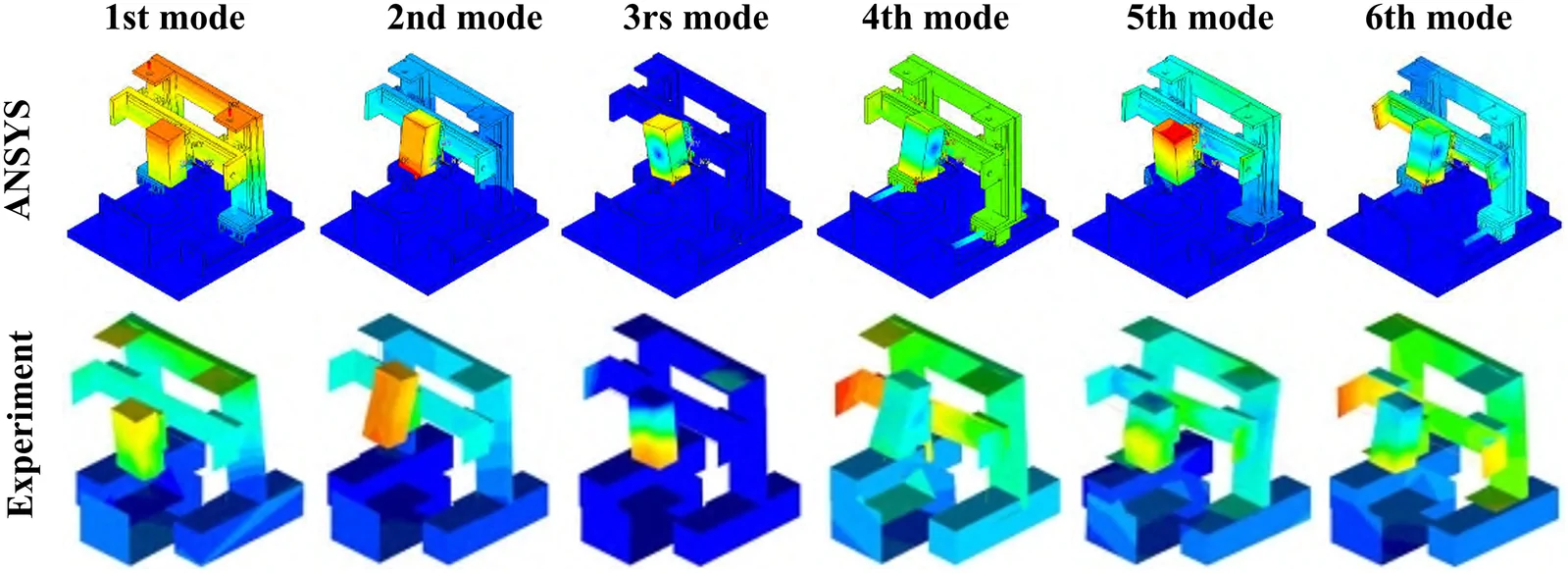
Qualitative comparison of reference-pose mode shapes.

The mode-shape images permit a qualitative correspondence check only. A numerical autoMAC matrix was not calculated because the underlying experimental mode-shape vectors were not available in the revision archive.

### 3.2. Pose-dependent predictions

Using the parametric finite element modeling method described in Section 2, a parametric finite element model of the five-axis machine tool analog specimen was established to predict its natural characteristics at different poses. The analog specimen used in these calculations was the same specimen used for finite element calibration and modal testing. Component interference limited the usable coordinate domain. The interference-constrained ranges adopted for the workspace calculations were X = 0–300 mm, Y = 200–350 mm, and Z = 100–300 mm. The A- and C-axis orientations were fixed at 0 degrees for the planar frequency surfaces in Fig 14.

Fig 15 presents the first three predicted natural frequencies on three orthogonal working planes. For the XY plane, Z was fixed at 200 mm and X and Y were sampled at 30 mm and 15 mm intervals, respectively. For the XZ plane, Y was fixed at 275 mm and X and Z were sampled at 30 mm and 20 mm intervals, respectively. For the YZ plane, X was fixed at 150 mm and Y and Z were sampled at 15 mm and 20 mm intervals, respectively. The rotary-axis orientations were A = 0 degrees and C = 0 degrees in all three planes. The first natural frequency varied mainly with the Y- and Z-axis coordinates. Because the first mode was dominated by forward tilting of the moving beam and column, changes in pose altered the assembled mass distribution, global bending configuration, effective lever arms, and locations of the equivalent joints. These configuration changes, rather than a gravity-induced modification of the material stiffness matrix, are consistent with the predicted first-mode frequency trend. The second and third modes were dominated by local spindle motion and therefore showed similar patterns within each working plane. The approximately symmetric trend with X reflects the symmetry of the gantry structure. These surfaces are numerical predictions from the calibrated model and were not independently confirmed by modal tests at multiple poses.

**Fig 15:**
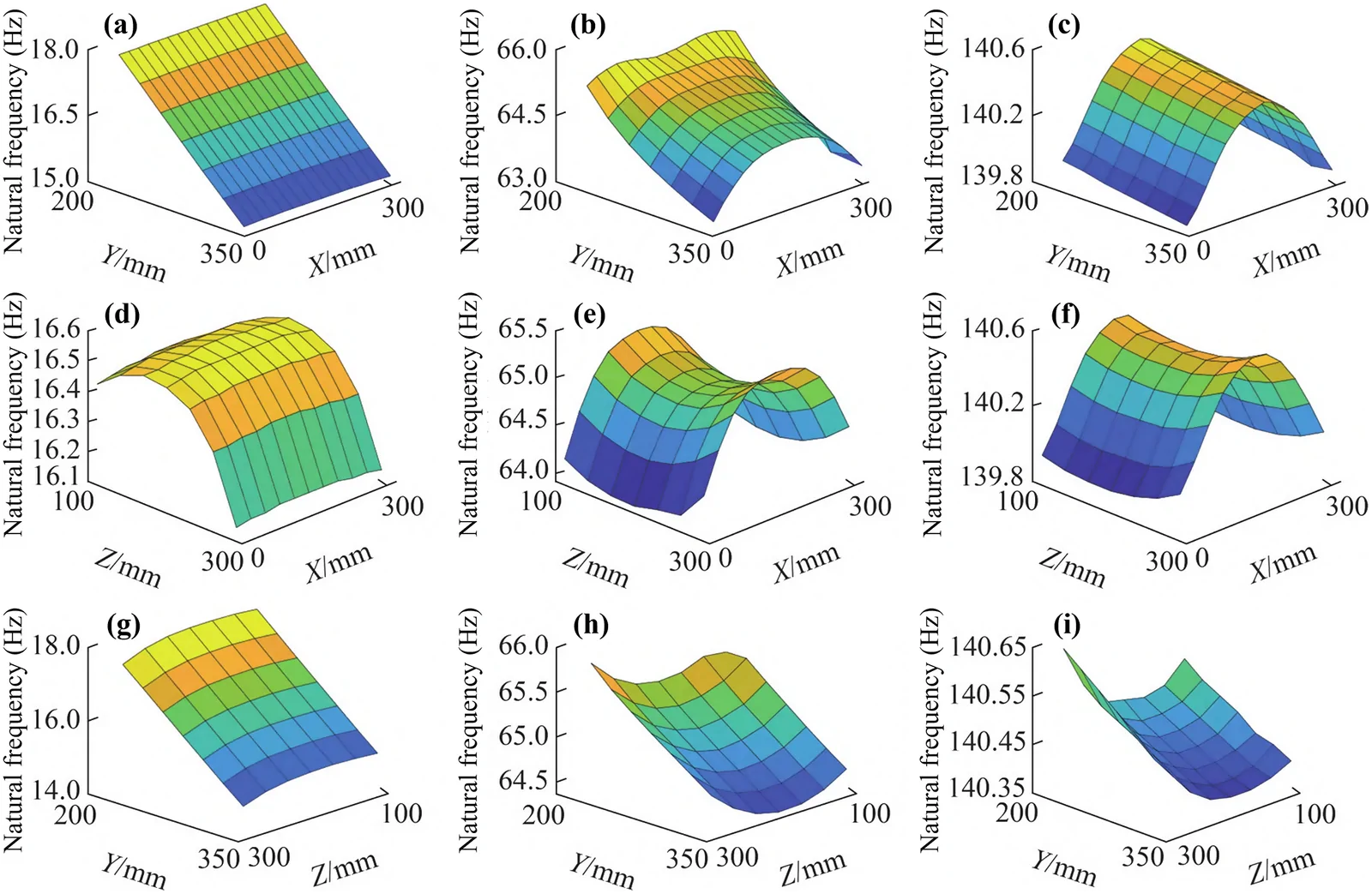
Variation patterns of predicted natural frequencies with pose changes. (a-c) XY plane at Z = 200 mm; (d-f) XZ plane at Y = 275 mm; (g-i) YZ plane at X = 150 mm. The sampling intervals were 30 mm along X, 15 mm along Y, and 20 mm along Z; A = 0 degrees and C = 0 degrees for all panels. Panels (a), (d), and (g) show the first mode; panels (b), (e), and (h) show the second mode; and panels (c), (f), and (i) show the third mode.

## 4. Discussion

The predicted first-mode trend is mechanically consistent with the changing mass distribution of the moving beam: increasing the beam height changes the global bending configuration and lowers the first natural frequency. The nearly parallel behavior of the second and third frequencies across working planes reflects their spindle-dominated local mode shapes. These findings support the use of parametric geometry updates for screening pose-sensitive regions of the workspace, but they do not establish experimental accuracy away from the reference pose.

The present formulation differs from approaches that combine position-dependent models with measurements at several machine positions [4,5,23]. Here, the twelve calibrated screw-nut and guideway-slider stiffness values are constant with pose, and damping is neglected. Accordingly, the model isolates changes caused by geometry, relative component location, and mass distribution, but it cannot reproduce load-dependent joint stiffness or position-dependent damping. This boundary should be considered when the model is used for design decisions or machining-stability prediction.

Four limitations are central. First, no formal mesh-convergence study was retained, so discretization uncertainty is not separately quantified. Second, the same single-pose modal frequencies were used for stiffness calibration and internal model checking; independent measurements at additional poses are needed to test workspace transferability. Third, representative FRFs, coherence functions, and numerical mode-shape vectors were unavailable, preventing direct assessment of signal quality and autoMAC. Fourth, the genetic-algorithm initialization bounds were not preserved. These limitations motivated the revised claim language: the model provides pose-dependent predictions and an efficient analysis framework, not a fully validated digital representation of the machine across its workspace.

## 5. Conclusions

This study developed a parametric finite element workflow for efficient calculation of pose-dependent natural characteristics of a five-axis machine-tool analog specimen. The model combines modular finite element construction, equivalent joint springs, genetic-algorithm stiffness calibration at one reference pose, and local coordinate-based updating of movable components and joints. At the calibration pose, the mean and maximum discrepancies for the first six natural frequencies were 3.29% and 8.78%, respectively. The workspace calculations predicted a reduction in the first natural frequency as the moving beam center of gravity rose, while spindle-dominated local modes displayed similar trends across working planes. Because the stiffness parameters were held constant and no independent multi-pose modal dataset was available, these results should be interpreted as model-based predictions. Multi-pose impact testing with archived FRFs, coherence functions, quantitative MAC metrics, and a documented mesh-convergence study is required before the model can be considered validated throughout the workspace.
